# Diagnosing a Fractured Pedunculated Osteochondroma Using Point-of-Care Ultrasound in the Emergency Department: A Case Report

**DOI:** 10.5811/cpcem.63912

**Published:** 2026-10-31

**Authors:** Jared Wilson, Martin Tremwel, Madeline Query

**Affiliations:** University of Illinois College of Medicine–Peoria, Department of Emergency Medicine, Peoria, Illinois

**Keywords:** osteochondroma, point-of-care ultrasound, pediatric, musculoskeletal ultrasound, case report

## Abstract

**Introduction:**

This case report discusses the use of point-of-care ultrasound (POCUS) in the diagnosis of a fractured pedunculated osteochondroma in an 8-year-old boy after minor trauma. Although osteochondromas are the most common benign bone tumors, especially in this age, historically only radiographs have been obtained in the emergency department to diagnose these benign bone tumors.

**Case Report:**

An 8-year-old boy reported ongoing pain for 2 months but had an acute onset worsening of his pain after minor trauma to the medial distal right femur. Point-of-care ultrasound was used to diagnose a fractured pedunculated osteochondroma prior to radiographic imaging.

**Conclusion:**

We demonstrated the practicality and ease of POCUS in assisting with the diagnosis of an osteochondroma and the subtle fracture of the base. Both POCUS and radiographic imaging may be beneficial when used in conjunction for the diagnosis of benign bone tumors in this age group.

## INTRODUCTION

Osteochondroma is the most common benign bone tumor, most commonly diagnosed in children younger than 20 years of age.^1^ They account for 20% to 50% of benign bone tumors and 9% of all bone tumors.^1^–^4^ There are two specific features of osteochondromas: the presence of cortical and medullary continuity; and the presence of a cartilage cap.^1^ While mostly painless, causes of pain include fracture, nerve impingement, or vascular compromise.^1^ Initial diagnostic study of choice is a radiograph while ultrasound can aid in the diagnosis and evaluation, particularly in measuring cartilage cap thickness.^1^ A cap thickness less than 2.0 cm in children and less than 1.5 cm in adults is reassuring and less likely to have malignant transformation.^2^ This presentation was typical, but we demonstrated use of point-of-care ultrasound (POCUS) in the initial diagnosis and were able to diagnose fracture and cortical irregularity which have historically been done solely with radiographs.

## CASE REPORT

### History

An 8-year-old boy presented to our emergency department (ED) for acute on chronic right knee pain. He initially reported two months of right knee pain with acute worsened symptoms the night before after bumping his medial knee with his elbow. He now reported a palpable bump. He was initially evaluated by his primary care clinician several months earlier and was diagnosed with growing pains.

The patient had no significant past medical or social history. His only allergy was to cephalexin. On physical exam, he was noted to have a palpable swollen/firm area on the right distal medial femur without evidence of overlying erythema, fluctuance or induration. This area was significantly tender to palpation. He maintained full range of motion of the knee.

### Diagnostic Imaging

Initial diagnostic imaging in this case was POCUS (Image 1). This demonstrated a hyperechoic density visualized on the right distal medial femur with likely step-off or defect consistent with fracture. Given the concern for cortical irregularity and possible bone lesion, further imaging was obtained, which included radiographs of the right knee in four views as well as a consultative ultrasound performed in the radiology suite.

Radiographs demonstrated a circumscribed exophytic bone lesion arising from the medial aspect of the distal femur measuring approximately 20 × 10 mm (Image 2). This was contiguous with adjacent cortex and trabeculae and characteristic for an osteochondroma. Seen best on the anteroposterior view, there appeared to be a fracture through the base of the osteochondroma with approximately 1 mm cortical step-off inferiorly. Consultative ultrasound demonstrated an exophytic osseous lesion arising from the distal medial femur, consistent with an osteochondroma (Image 3).

With the above findings of osteochondroma with fracture as seen on both plain radiographs and ultrasound, the pediatric orthopedics team recommended outpatient follow-up for further discussion of management. He was discharged from the ED in stable condition, able to bear weight as tolerated. He was seen in an orthopedic clinic two days later where they discussed management options including surgical excision. The patient and his mother elected to undergo surgical excision and underwent successful surgical removal of the osteochondroma two months later.

*CPC-EM Capsule*

What do we already know about this clinical entity?

*Osteochondromas are common benign bone tumors in children; fracture is a recognized complication that can cause acute pain.*

What makes this presentation of disease reportable?

*This case demonstrates a novel use of point-of-care ultrasound (POCUS) to diagnose a fractured distal femoral osteochondroma after minor trauma in a child.*

What is the major learning point?

*POCUS can identify cortical irregularity and fracture of an osteochondroma and may complement radiographs in the emergency department.*

How might this improve emergency medicine practice?

*POCUS may help emergency physicians rapidly identify bony lesions and suspected fracture, guiding radiography and specialty follow-up.*

## DISCUSSION

This case demonstrates the ability of POCUS to assist in the diagnosis of fractured osteochondromas in the ED. Although plain radiographs are still the standard imaging modality for initial diagnosis, ultrasound has been shown to be an effective supplemental tool. In osteochondroma, ultrasound is typically used to evaluate the cartilaginous cap rather than radiographs as initial diagnostic imaging.^1^ This case was a typical presentation of classic osteochondroma but had an atypical fracture history with only very minor trauma to the area. Ultrasound enabled visualization of the cortical irregularity concerning for fracture pathology, which was able to be fully visualized on plain radiographs.

No similar case reports were identified in our literature review. Existing literature discusses the utility of POCUS for fractures as a reasonable option when radiographs are unavailable or to limit radiation exposure.^5^ However, despite the benefits of ultrasound, its use can be limited because it is operator dependent.^5^ Specific to osteochondroma, POCUS does not allow visualization of the cartilage cap when it is inwardly oriented or deep in soft tissue.^5^

## CONCLUSION

An 8-year-old boy presented with a typical presentation of osteochondroma of the distal right femur. His acute onset of worsened pain after minor trauma was likely related to fracture through the stalk of the lesion, which was able to be visualized on POCUS as well as confirmed on radiographic imaging. He followed up with an orthopedic specialist and underwent successful excision two months after initial diagnosis in the ED. The case demonstrates that POCUS can be an effective tool in facilitating the diagnosis of benign bone tumors.

## Figures and Tables

**Image 1:**
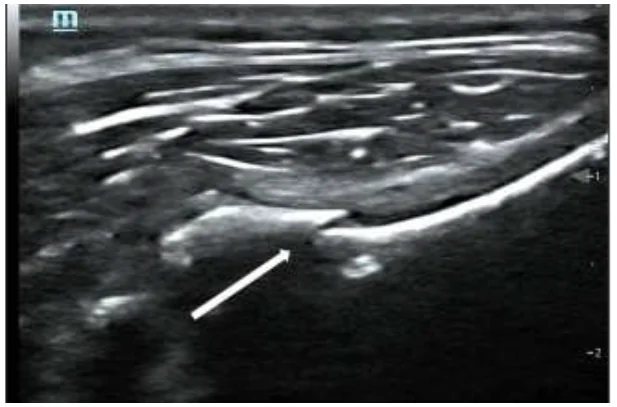
Point-of-care musculoskeletal ultrasound using a linear probe in long axis demonstrates a hyperechoic step-off (white arrow) consistent with distal medial femur fracture.

**Image 2:**
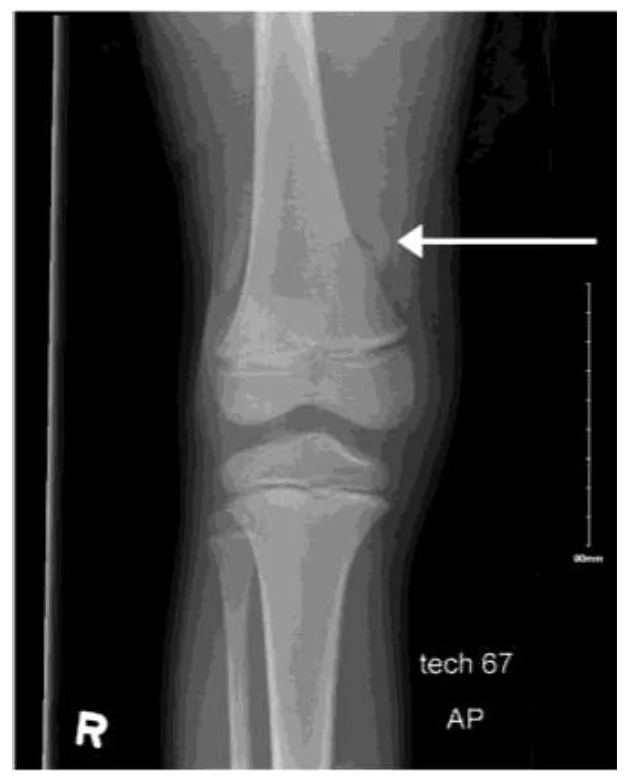
Right knee radiograph demonstratesa bone lesion arising from the medial aspect of the distal femoral diametaphysis; appearance is characteristic of an osteochondroma (white arrow).

**Image 3:**
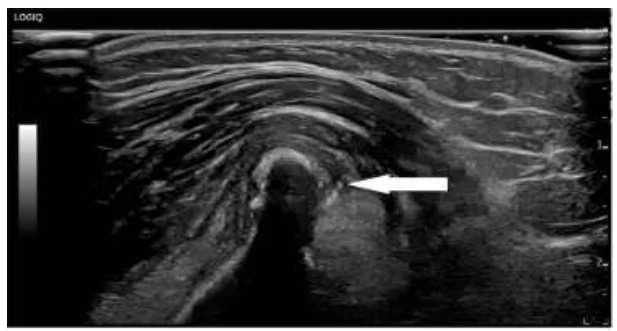
Radiologist-performed ultrasound of an exophytic osseous lesion arising from the distal right medial femur, consistent with an osteochondroma (white arrow).
